# Egypt's second wave of coronavirus disease of 2019 pandemic and its impact on patients with inflammatory bowel disease

**DOI:** 10.1002/jgh3.12551

**Published:** 2021-05-06

**Authors:** Safaa R Askar, Mohamed A Sakr, Waleed H A Alaty, Ahmed F Sherief, Heba Rashad, Mohamed Eltabbakh

**Affiliations:** ^1^ Tropical Medicine Department, Faculty of Medicine Ain Shams University Cairo Egypt

**Keywords:** COVID‐19, inflammatory bowel disease, pandemic, psychological stress, second wave

## Abstract

**Background and Aim:**

After a decrease of COVID‐19 cases in summer, Europe faced the appearance of a COVID‐19 second wave. IBD patients are more vulnerable to various emotional and psychological stresses than normal individuals. The aim of this study explored the emotional state, perception, and coping strategies of patients with IBD during the COVID 19 pandemic period.

**Methods:**

A cross‐sectional study was conducted between 15 November and 15 December 2020. Patients presented to IBD Study Group Clinic, Department of Tropical Medicine, Ain Shams University Hospitals. The study included 105 IBD patients. A predesigned questionnaire was used that focused on patients’ knowledge of the COVID‐19 pandemic, and how it influenced patient care. Patient demographics, disease characteristics, and medication type were analyzed.

**Results:**

We found 10.5% of patients stopped or delayed their medications owing to the COVID‐19 pandemic second wave and 61% reported that their clinic visits were affected. Seven patients were tested, and two patients reported having been diagnosed with COVID‐19, and 18 patients reported having relatives diagnosed with COVID‐19.

**Conclusion:**

A considerable number of patients with IBD had an interruption to their care because of the second wave COVID‐19 pandemic. So, patients with IBD should take attention before, during, and after such pandemics to avoid undesirable disease‐related outcomes.

## Graphical abstract

During COVID‐19 second wave, inflammatory bowel disease (IBD) patients are at risk of pandemic‐related care interruptions. So, attention should be paid to IBD patients before, during, and after any infectious epidemics.

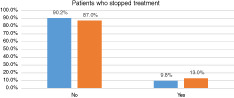

## Introduction

Coronavirus disease triggered by Severe Acute Respiratory Syndrome Coronavirus (SARS‐CoV‐2) hit Hubei province by the end of 2019 and has turned into a global pandemic.^1^ Humanity faces its greatest challenge in the last hundred years as the 2019 coronavirus disease outbreak has caused about 17 million cases confirmed worldwide.^2^

Europe has faced the emergence of a possible second wave of COVID‐19 infection since the end of July 2020, following a decline in the detected cases in summer. The suspected cases of the disease continued to increase, suggesting the start of a new wave of outbreaks of infectious diseases.^3^

Numerous studies have concluded that COVID‐19 has a fatality rate ranging between 7.2% and 67% and is mainly affected by age, underlying diseases, and pneumonia severity.^4^, ^5^, ^6^ However, it has been reported that younger individuals with no known underlying diseases have also contracted the infection and became critically ill, with reported hospitalization rates ranging from 20.7% to 31%.^7^ Besides, asymptomatic patients have been reported to be potentially able to transmit the disease through close contact during the incubation period, which is the primary mechanism involved in rapid and widespread community transmission.^8^

Both experimental and clinical data have shown that SARS‐CoV‐2 can affect people of all ages, in particular older adults and those with underlying diseases.^9^ Inflammatory bowel disease (IBD) is an immune‐related disease that is often treated with immune modification or immunosuppressive therapy to control the symptoms and cure the mucosa.^10^ IBD patients are more vulnerable than normal individuals to different emotional and psychological stresses.^11^, ^12^ Medications used in such treatments could even make patients more prone to multiple infections, which may become a critical threat for patients and healthcare professionals during an infectious pandemic.^13^

In addition to the general preventive measures widely advised during the current pandemic, hospitals around the world have also taken several restrictive measures, including rescheduling of clinical visits, endoscopic procedures, and infusion appointments, all of which can be alarming for patients with IBD and potentially distressing. Therefore, understanding the degree of IBD patients' fear, anxiety, and overall perception of a pandemic can provide critical insights that would help doctors deliver better clinical and psychological support during such times to prevent any inadequate coping strategies that are substantially linked to poor patient outcomes.^14^

Naturally, IBD patients alternately undergo remission and relapse periods that involve regular medication and follow‐up. However, locking and limitations for travel are introduced in areas hit hard by COVID‐19. As a result, IBD patients cannot be monitored as scheduled and may have limited or no access to the medicines they need. The disease may relapse or worsen if patients are cut off from their medication.^15^

The aim of this study explored the emotional state, perception, and coping strategies of patients with IBD during the COVID 19 pandemic period. It would help them to combat future threats that are similar.

## Methods

### Subjects

**Figure 1:**
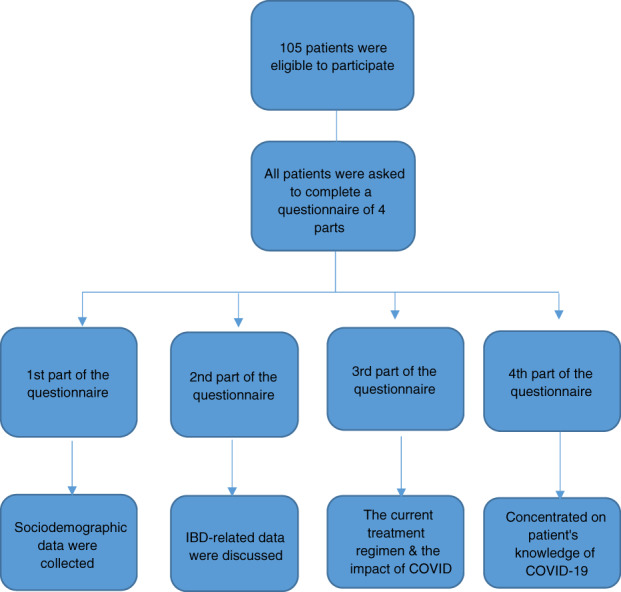
Flow chart of the study.

Our study comprised 105 IBD patients linked to the IBD Study Group Clinic, Department of Tropical Medicine, Ain Shams University Hospitals. IBD was diagnosed based on conventional disease criteria. All patients were asked through telephone to complete a questionnaire (Fig. 1) between 15 November and 15 December 2020 during the second wave of the COVID‐19 epidemic.

### Design of the current study

The clinical study was cross‐sectional and observational in nature. Demographic and social details from IBD patients have been collected. The Medical Ethical Review Committee, Ain Shams Medical College, Ain Shams University, Cairo, Egypt, approved this study.

### Questionnaires

In the first part of the questionnaire, data on sociodemographic data of patients were included, covering mainly age, gender, marital status, and place of residence. Details of IBD‐related data involving subtypes (Crohn's disease and ulcerative colitis) were discussed in the second part. In the third part of the questionnaire, the current treatment regimen and the impact of the COVID 19 pandemic on patient adherence were addressed. The fourth part concentrated on patients' knowledge of the COVID 19 pandemic and how patient care was affected.

### Statistical analysis

Data were presented as the mean ± SD. Differences between groups were assessed using a *t*‐test or anova. To determine the structural relationship between the measured variables, Pearson's correlation analysis and multivariate analysis were employed. Statistical analysis was conducted using SPSS statistical package.

## Results

### Subjects

One hundred and five patients with inflammatory bowel disease were included and they were, on average, 33.2 years old. Forty‐nine men and 56 women were present. There were 77 urban and 28 rural patients, 53 single patients, 48 married, and 4 divorced patients (Table 1).

**Table 1:** Baseline characteristics of the study

	Mean/*n*	SD/%	Median (interquartile range)	Range
Age	33.2	11.0	32 (25–40)	(12–76)
Sex				
Female	56	53.3%	—	—
Male	49	46.7%	—	—
Residency				
Urban	77	73.3%	—	—
Rural	28	26.7%	—	—
Marital status				
Single	53	50.5%	—	—
Married	48	45.7%	—	—
Divorced	4	3.8%	—	—
IBD				
Ulcerative colitis (UC)	82	78.1%	—	—
Crohn's disease	23	21.9%	—	—
Mayo Score UC (*n* = 82)	8.4	2.6	8 (7–10)	(4–12)
Crohn's disease activity index (*n* = 23)	238.84	62.29	251 (180–275)	(159–413)

Mayo Score is an index for severity of ulcerative colitis.

### Current status of patients

There were 23 Crohn's disease patients in our series; 82 ulcerative colitis (UC) patients (Table 1). The main medications taken by patients with IBD were 70.5% aminosalicylic acid (74/105), 34.3% glucocorticoid (36/105), 66.7% azathioprine (70/105), and 20% biological agents (21/105) (Table 2).

**Table 2:** Different types of treatment

Type of treatment	*n*	%
Oral mesalamine		
No	31	29.5
Yes	74	70.5
Topical mesalamine		
No	101	96.2
Yes	4	3.8
Oral steroids		
No	69	65.7
Yes	36	34.3
Topical steroids		
No	103	98.1
Yes	2	1.9
Azathioprine		
No	35	33.3
Yes	70	66.7
Biological therapy		
No	84	80.0
Yes	21	20.0
Surgical intervention		
No	98	93.3
Yes	7	6.7

### Medication inaccessibility is a serious issue for patients with IBD

**Figure 2:**
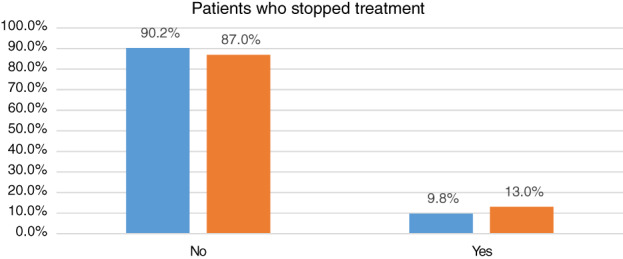
Continuation and discontinuation of medications in inflammatory bowel disease patients during the COVID‐19 epidemic. (a) The number of UC patients who continued and discontinued their medications. (b) The number of CD patients who continued and discontinued their medications. 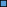 UC, ulcerative colitis; 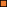 CD, Crohn's disease

10.5% of patients (11/105) reported that because of the COVID 19 pandemic second wave in Egypt, they stopped their medications or delayed them (Table 3). Patients who stopped treatment were divided into three Crohn's disease patients and eight ulcerative colitis patients (Fig. 2).

**Table 3:** Perceptions of COVID‐19 among patients with inflammatory bowel disease (IBD)

	*n*	%
How dangerous is COVID‐19		
Somewhat	19	18.1
Very	49	46.7
Extremely	37	35.2
Were you tested for COVID 19		
No	98	93.3
Yes	7	6.7
Diagnosed?		
No	103	98.1
Yes	2	1.9
Did COVID 19 disturb your clinic visits		
No	41	39.0
Yes	64	61.0
Any relative diagnosed with COVID 19		
No	87	82.9
Yes	18	17.1
IBD more prone?		
No	13	12.4
Yes	92	87.6
Did you stop TTT?		
No	94	89.5
Yes	11	10.5

### Perceptions of COVID 19 among IBD patients

The bulk of the patients perceive COVID‐19 as either “very dangerous” (46.7%) or “extremely dangerous” (35.2%). Eighty‐seven percent of the patients believed that IBD patients were much more likely than the general population to contract COVID 19, and 61% revealed that their clinic visits were affected by the pandemic (Table 3).

### COVID 19 in IBD patients

Of the seven patients tested, two patients reported having been diagnosed with COVID 19. Eighteen patients reported having family members diagnosed with COVID 19 (Table 3). Both patients were female, were diagnosed with ulcerative colitis, and had no comorbidity (Table 4).

**Table 4:** A description of the two patients who reported being diagnosed with COVID‐19

	Age	Gender	Residence	Disease	Medications	Comorbidities
1	17	Female	Urban	Ulcerative colitis (UC)	Glucocorticoids, AZA, Aminosalicylate	None
2	69	Female	Urban	UC	Aminosalicylate	None

## Discussion

Since the WHO has declared a world health emergency to COVID 19 pandemic, physicians treating patients with IBD were recommended to take strict precautions against the virus, delaying non‐urgent endoscopic procedures and taking into account the risks of immunosuppressive drugs.^16^

All these measures may possibly trigger or aggravate mood disturbances in IBD patients. As a consequence, 87% of patients assumed they were more likely to contract COVID 19 than the general population, and 61% confirmed that their clinic visits were affected by the pandemic.

Drug inaccessibility is a serious issue for patients with IBD. In the current study, 10.5% of patients (11/105) reported difficulty accessing medicines during the COVID‐19 epidemic because of home quarantine. However, Huan Wang et al. reported that two‐thirds of IBD patients in Wuhan had difficulty accessing medicines, and half of them had discontinued their medicines.^4^

A similar cross‐sectional study by Mosli et al. showed that 30.7% of patients reported either stopping or delaying their drug during the pandemic. The two primary reasons for this behavior were the belief that the drugs are predisposed to infection (35.5%) and the fear of visiting the hospital or doctor's office during the outbreak (27.3%).^17^

The perception of COVID‐19 was either “very dangerous” (46.7%) or “extremely dangerous” (35.2%) among patients. These findings agreed with those of Mosli et al. who noted that the perception by many two‐thirds of participants was that the COVID 19 virus was extremely harmful, with about one‐third of patients assumed that they were at a higher risk of becoming infected due to their illness.^17^

In our study, seven patients were tested for COVID‐19 and two patients reported a positive diagnosis of COVID‐19. Both patients were female, had ulcerative colitis diagnosed, and had no comorbidities. These findings agreed with those of Mosli et al. (2020) who reported that 30 patients had been tested and 6 patients had been diagnosed with COVID‐19. There were also no comorbidities in the six patients.^17^

## Conclusion

A significant percentage of patients have reported pandemic‐related interruptions in their care. Consequently, to prevent unwanted disease‐related outcomes, attention should be paid to patients with IBD before, during, and after these epidemics.

This study helps doctors to understand IBD patients' physical and mental conditions and to manage IBD patients better during the ongoing pandemic.

## References

[1] World Health Organization Coronavirus 2020. Available from URL https://www.who.int/health-topics/coronavirus#tab=tab_1.

[2] Coronavirus disease (COVID‐19) situation reports. Available from URL https://www.who.int/emergencies/diseases/novel‐coronavirus‐2019/situation‐reports [Internet].

[3] Bontempi E . The Europe second wave of COVID‐19 infection and the Italy "strange" situation. Environ. Res. 2021; 193: 110476. 33221311 10.1016/j.envres.2020.110476 PMC7674970

[4] Onder G , Rezza G , Brusaferro S . Case‐fatality rate and characteristics of patients dying in relation to COVID‐19 in Italy. JAMA. 2020; 323(18): 1775–6. 32203977 10.1001/jama.2020.4683

[5] Arentz M , Yim E , Klaff L *et al*. Characteristics and outcomes of 21 critically ill patients with COVID‐19 in Washington state. JAMA. 2020; 323(16): 1612–4. 32191259 10.1001/jama.2020.4326 PMC7082763

[6] Young BE , Ong SW , Kalimuddin S *et al*. Epidemiologic features and clinical course of patients infected with SARS‐CoV‐2 in Singapore. JAMA. 2020; 323(15): 1488–94. 32125362 10.1001/jama.2020.3204 PMC7054855

[7] CDC COVID‐19 Response Team . Severe outcomes among patients with coronavirus disease 2019 (COVID‐19)—United States, February 12–March 16, 2020. MMWR Morb. Mortal. Wkly Rep. 2020; 69: 343–6. 32214079 10.15585/mmwr.mm6912e2 PMC7725513

[8] Rothe C , Schunk M , Sothmann P *et al*. Transmission of 2019‐nCoV infection from an asymptomatic contact in Germany. N. Engl. J. Med. 2020; 382: 970–1. 32003551 10.1056/NEJMc2001468 PMC7120970

[9] Kirchgesner J , Lemaitre M , Carrat F , Zureik M , Carbonnel F , Dray‐Spira R . Risk of serious and opportunistic infections associated with treatment of inflammatory bowel diseases. Gastroenterology. 2018; 155: 337–346.e10. 29655835 10.1053/j.gastro.2018.04.012

[10] IOIBD Update on COVID‐19 for patients with Crohn's disease and ulcerative colitis. 2020 Available from URL https://www.ioibd.org/ioibd‐update‐on‐covid19‐for‐patients‐with‐crohns‐disease‐and‐ulcerative‐colitis/ [Last accessed on April 20 2020].

[11] Kubesch A , Boulahrout P , Filmann N , Blumenstein I , Hausmann J . Real‐world data about emotional stress, disability and need for social care in a German IBD patient cohort. PLoS One. 2020; 15: e0227309. 31899780 10.1371/journal.pone.0227309 PMC6941800

[12] Gamwell KL , Roberts CM , Espeleta HC *et al*. Perceived stigma, illness uncertainty, and depressive symptoms in youth with inflammatory bowel disease: the moderating effect of mindfulness. Psychol. Health Med. 2020; 25(9): 1037–48. 31941362 10.1080/13548506.2020.1714062

[13] Toruner M , Loftus EV Jr , Harmsen WS *et al*. Risk factors for opportunistic infections in patients with inflammatory bowel disease. Gastroenterology. 2008; 134(4): 929–36. 18294633 10.1053/j.gastro.2008.01.012

[14] Chao CY , Lemieux C , Restellini S *et al*. Maladaptive coping, low self‐efficacy and disease activity are associated with poorer patient‐reported outcomes in inflammatory bowel disease. Saudi J. Gastroenterol. 2019; 25: 159–66. 30900609 10.4103/sjg.SJG_566_18 PMC6526742

[15] Wang H , Lei T , Li Y *et al*. The symptoms and medications of patients with inflammatory bowel disease in Hubei Province after COVID‐19 epidemic. J Immunol Res. 2020; 2020: 2847316. 33062719 10.1155/2020/2847316 PMC7547343

[16] Danese S , Ran ZH , Repici A *et al*. Gastroenterology department operational reorganisation at the time of covid‐19 outbreak: an Italian and Chinese experience. Gut. 2020; 69(6): 981–3. 32299837 10.1136/gutjnl-2020-321143

[17] Mosli M , Alourfi M , Alamoudi A *et al*. A cross‐sectional survey on the psychological impact of the COVID‐19 pandemic on inflammatory bowel disease patients in Saudi Arabia. Saudi J. Gastroenterol. 2020; 26: 263–71. 32567580 10.4103/sjg.SJG_220_20 PMC7739990

